# Epidemiology and control of hepatitis C virus infection in Brunei Darussalam: a retrospective cohort study

**DOI:** 10.1016/j.ijregi.2025.100818

**Published:** 2025-11-29

**Authors:** Kai Shing Koh, Justin Wong, Liling Chaw

**Affiliations:** 1 Brunei Centre for Disease Control and Prevention, Department of Environmental Health Services, Ministry of Health, Bandar Seri Begawan, Brunei Darussalam; 2 PAPRSB Institute of Health Sciences, Universiti Brunei Darussalam, Bandar Seri Begawan, Brunei Darussalam

**Keywords:** Hepatitis C virus, Epidemiology, Treatment, Mortality, Brunei

## Abstract

**Objectives:**

We describe the epidemiology of hepatitis C virus (HCV) cases in Brunei Darussalam and evaluate factors associated with its treatment outcomes.

**Methods:**

A retrospective cohort study was conducted using data from two national databases between January 2013 and December 2022.

**Results:**

We identified 801 anti-HCV-positive cases. Although incidence remained relatively stable, prevalence rates increased from 10.1 to 48.7 per 100,00 population. Among those with detectable HCV RNA, treatment was initiated in 52.3% (n = 239). Locals (adjusted odds ratio [aOR] = 2.42, 95% confidence interval [CI] 1.16, 5.36) and age (30-39 years [aOR = 2.41, 95% CI 1.17, 5.07], 40-44 years [aOR = 2.55, 95% CI 1.19, 5.58], and 50-54 years [aOR = 2.76, 95% CI 1.25, 6.24) were associated with treatment initiation. Among those who completed treatment (69.5%, n = 166), 64.5% (n = 107) achieved sustained virologic response. HCV-related complications at baseline were recorded in 7.4% (n = 59) of all cases, particularly among those aged ≥50 years (aOR = 3.32, 95% CI 1.93, 5.79). HCV-related deaths occurred in 36.4% (n = 47), among which 44.7% (n = 21) had HCV-related complications at baseline.

**Conclusions:**

An increasing HCV prevalence (likely due to case management processes and patient-related factors) and the high proportion of deaths with baseline HCV-related complications suggest the need to strengthen HCV screening and management in Brunei.

## Highlights

• We report an increasing prevalence of hepatitis C virus (HCV) cases in Brunei.
• This is possibly due to declining treatment initiation and completion rates.
• Almost half of all HCV deaths had baseline HCV-related complications.
• Suggests need to improve primary and secondary prevention activities for HCV.

## Introduction

Globally, the World Health Organization (WHO) estimated about 50 million people worldwide were living with hepatitis C virus (HCV) infection, with about 1.0 million new infections and about 220,000 deaths in 2022 [1]. HCV accounted for 15.3 million global disability-adjusted life years from acute hepatitis (1.7%), cirrhosis (79.5%), and liver cancer (18.8%) [2]. Among the countries in the WHO Western Pacific Region, HCV accounted for 26% of all liver cancer deaths as well as 27% of deaths due to liver cirrhosis and other chronic liver diseases [3,4]. In 2019, 25% of the top 20 countries globally for HCV-related deaths are within the WHO Western Pacific Region [3,4].

Treatment for HCV infection initially involves a combination of pegylated interferon-alpha (in the form of weekly injections) and daily ribavirin for 24-48 weeks [5]. It was later replaced with pan-genotypic direct-acting antivirals (DAAs) [1]. The main aim of treatment is to achieve sustained virologic response (SVR), defined by the absence of detectable HCV RNA 24 weeks post-treatment with pegylated interferon or 12 weeks post-treatment with DAAs. SVR attainment has been associated with improvements in all-cause mortality, liver-related mortality, and the development of hepatocellular carcinoma, as well as other hepatic-related complications [6,7].

Brunei Darussalam (population 430,000) is a small Southeast Asian country with a Markov model-estimated HCV prevalence of 0.33% in 2022 [8]. The country is included in the long list of countries with no HCV prevalence estimates regionally and globally, a threat recognized by the WHO [9,10]. Although local research on HCV was previously conducted, these studies were primarily focused on treatment response, genotyping, or prevalence among a subgroup of patients [[11], [12], [13]]. To date, no studies have described the prevalence and natural history of HCV in Brunei.

Thus, this study aims to describe the epidemiological and control indicators of HCV cases in Brunei Darussalam. Aggregating available national-level data from national surveillance and electronic medical record system databases, we specifically aim to (i) determine the incidence and prevalence of HCV cases in the country, (ii) identify the common possible sources of HCV transmission, (iii) identify the determinants of HCV treatment initiation and cure, and lastly, (iv) and determine the factors associated with HCV-related complications and deaths.

Our study findings could inform stakeholders about the scale of this issue and identify ways to address it in the national context. Operationally, our findings could enable decision-makers to estimate the number of people requiring HCV treatment for HCV resource planning and allocation, as well as to assess the needs for screening and testing in target population groups.

## Methods

### Study design and setting

We conducted a retrospective cohort study that included all diagnosed HCV cases in Brunei Darussalam from January 2013 to December 2022 (10-year period). An HCV case is defined as one whose serum sample tested positive for antibodies against HCV (anti-HCV). We have included all HCV cases regardless of existing co-morbidities or presence of co-infection with HIV or hepatitis B virus (HBV).

### Case reporting, testing, and investigation

In Brunei, HCV screening via the detection of anti-HCV in blood samples is performed for (i) occupational health screening among health care professionals, (ii) pre-employment medical fitness among government employees and foreign workers, (iii) end-stage renal failure patients undergoing hemodialysis, and (iv) donated blood and blood products [11,12]. HCV screening may be conducted in clinical settings for patients presenting with suspected liver pathology and for those with cancers before initiating chemotherapy.

HCV is a notifiable disease under Brunei’s Infectious Disease Act since 2003 [14]. Health care professionals from both public and private health facilities in the country are mandated to report all positive anti-HCV cases to the Brunei Centre for Disease Control and Prevention (Brunei CDC), Ministry of Health. Once reported, case counselling and active case investigations are conducted by Brunei CDC to identify possible sources of transmission as well as any high-risk close contacts. All HCV cases will be referred to the Hepatology Clinic for further evaluation and management [11]. Only HCV cases with detectable HCV RNA (indicating chronic infection) will be offered HCV treatment.

For identified close contacts, the source case will usually provide their details and relay the information for blood HCV screening. Afterward, the results will be tracked by the Brunei CDC. If negative, no follow-up will be required. However, if positive for anti-HCV, they will be contacted and counselled as per a confirmed HCV case. In instances where the close contacts did not attend HCV screening, the source case will be contacted and reminded. However, it is voluntary whether the source cases inform their close contacts and whether their close contacts agree to undergo HCV screening.

### Data collection and case identification

To ensure completeness in case detection, data for this study were obtained from two sources: EVYDENCE (2013-2022) and the HCV registry (2017-2022). The former is a digital platform that integrates data from several national databases, primarily the national e-health records system, the Brunei Health Information Management System database (Bru-HIMS). Bru-HIMS is a one-patient-one-record system linking primary and secondary care clinical records, laboratory and radiological investigations that cover >99% of the population, and was first introduced in government health centers and hospitals in 2013 [15]. The HCV registry is a manual, offline registry maintained by the Brunei CDC and contains all notified HCV cases in the country.

Data collected from EVYDENCE include socio-demographics (age, gender, nationality, ethnicity, district of residence), year of first anti-HCV positive test, evidence of HCV treatment initiation and completion, HCV RNA level 24- or 12-weeks post treatment, HCV-related complications at baseline, and HCV-related deaths. For HCV-related complications, further information was collected to identify the type of complications, which include chronic liver disease, liver fibrosis and/or cirrhosis, liver failure, and liver carcinoma. Similarly, further data were collected for the type of HCV-related deaths, including liver cirrhosis, liver failure, and liver carcinoma. Data collected from the HCV registry include socio-demographics (age, gender, nationality, ethnicity, and district of residence), source of HCV acquisition, and the year the individual was registered as a positive HCV case.

### Data analysis

First, descriptive statistics were used to report the socio-demographics of all HCV cases in the study. Using the WHO monitoring and evaluation framework for viral hepatitis as a guide, we reported our outcomes of interest using indicators related to (i) treatment and care, including HCV treatment initiation and completion, and SVR attainment, (ii) morbidity, including incidence and prevalence, and HCV-related complications at baseline, and (iii) mortality, including HCV-related deaths [16].

We calculated incidence based on the number of new HCV cases during the study period. Annual prevalence was calculated by adding the total number of cases with detectable HCV RNA by the end of the previous year to the number of new cases with detectable HCV RNA in the current year, minus the number of deceased and cured cases from the current year. Paucity of data before 2013 means we could not estimate HCV prevalence in 2013. Both incidence and prevalence were reported as rates per 100,000 population.

Patients were considered to have initiated treatment when antiviral therapy for HCV had been prescribed on Bru-HIMS. Treatment completion was determined when an HCV RNA test was conducted post-treatment to evaluate for SVR. Patients were defined as having attained SVR if HCV RNA was undetectable either for 24 weeks or more post-treatment with pegylated interferon or for 12 weeks or more post-treatment with DAAs. Patients with HCV-related complications (such as liver cirrhosis, liver failure, and liver carcinoma) within 1 year of their HCV diagnosis were regarded as having HCV-related complications at baseline. HCV-related deaths refer to patients who died due to HCV-related causes, including liver cirrhosis, liver failure, and liver carcinoma.

Chi-square, Fisher’s exact, and Mann-Whitney’s tests were performed to determine any statistical differences between cases who were (and were not) tested for HCV RNA and between those who had (and did not have) HCV RNA detected. Logistic regression analyses were conducted to determine the association between independent variables (socio-demographic factors and year of HCV diagnosis) with several dependent variables, namely, HCV treatment initiation, HCV treatment completion, SVR attainment, HCV-related complications at baseline, and HCV-related deaths.

The total number of HCV cases differed for the logistic regression analyses in this study, as those with missing data were removed by default. To avoid reporting bias, the overall proportions were reported based on the total identified cases. All analyses were performed using R (ver. 4.2.3) [17]. A *P*-value of <0.05 was considered statistically significant.

## Result

**Figure 1:**
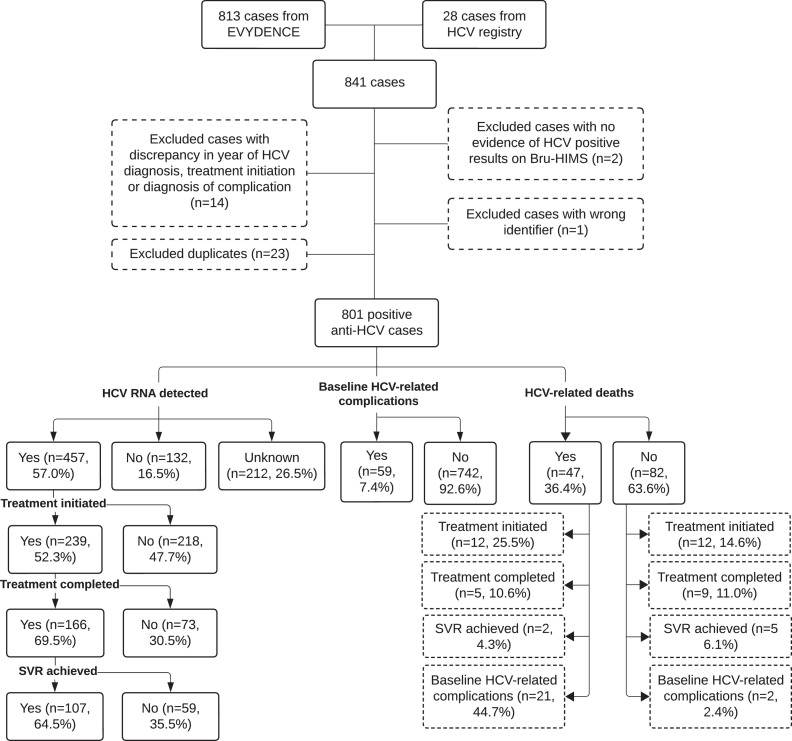
Study flow chart. Baseline HCV-related complications: patients who were found to have HCV-related complications (including liver cirrhosis, liver failure, and liver carcinoma) within 1 year of their HCV diagnosis; HCV-related deaths: patients who died due to HCV-related causes (including liver cirrhosis, liver failure, and liver carcinoma). HCV, hepatitis C virus; SVR, sustained virologic response.

A total of 841 HCV cases were identified from EVYDENCE (n = 813) and the HCV registry (n = 28) between January 2013 and December 2022 (10 years) and January 2017 to December 2022 (6 years), respectively. Duplicate records (n = 23), records with discrepancies in year of diagnosis and date of HCV treatment initiation or diagnosis of complication (n = 14), one case with a wrong personal identifier (n = 1), and patients with no evidence of HCV positive results in Bru-HIMS (n = 2) were excluded from the study. The final dataset consisted of 801 HCV cases (Figure 1).

### Incidence and prevalence

**Figure 2:**
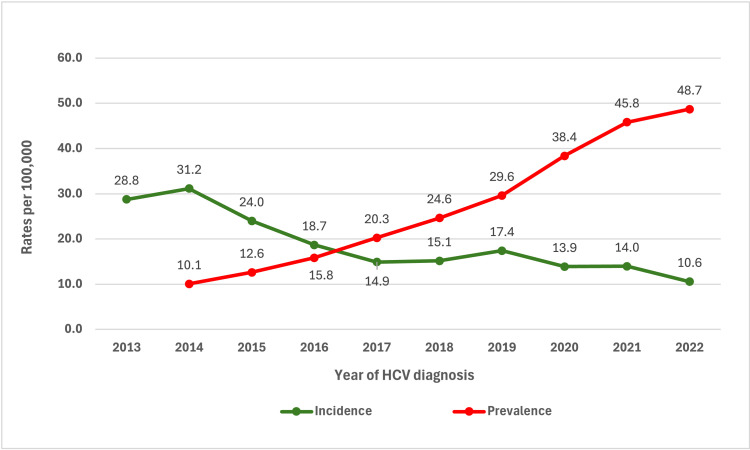
Annual HCV incidence and prevalence from 2013 to 2022, Brunei. HCV, hepatitis C virus.

The annual incidence rates of HCV cases were the highest in 2013 and 2014 at 28.8 and 31.2 per 100,000 population, respectively (Figure 2). It then trended downward and remained stable at under 20.0 per 100,000 population, with a slight decline after 2019. On the other hand, the annual prevalence rate increased gradually from 10.1 per 100,000 population in 2014 to 48.7 per 100,000 population in 2022.

### Case characteristics

The median age of the HCV cases was 44 years (interquartile range [IQR] 35-51; range 0–83), and males comprised most of the cases (662, 82.6%; Table 1). Locals (n = 691, 86.3%) made up a large proportion of the cases. The source of HCV acquisition included mother-to-child transmission (n = 1, 0.1%) and sexual intercourse (n = 1, 0.1%); however, the source for most of the cases was either unknown or not available (n = 799, 99.8%). Among these HCV cases, 457 (57.0%) had detectable HCV RNA, whereas 212 (26.5%) were not tested. There were no significant differences between patients who had HCV RNA detected and those who did not (Table 1), except for the year of diagnosis where the proportion of patients with HCV RNA detected was higher in 2020-2022 (*P* <0.001). Patients who did not have HCV RNA tested are significantly younger (*P* = 0.001) and are local residents (*P* <0.001; Supplementary Table S1). In addition, the proportion of patients who did not get tested for HCV RNA was higher in 2020-2022 (*P* = 0.004).

**Table 1:** Characteristics of HCV cases during the study period (2013-2022), Brunei.

Patient characteristics		Overall^a^ (%)	HCV RNA detected n (%)	HCV RNA not detected n (%)	*P*-value
**Total**		801 (100)	457 (57.0)	132 (16.5)	
**Median age (Interquartile range; range)**		44 (35-51; 0-83)	44 (37-51; 6-77)	47 (40-52; 0-76)	0.046
**Age group (years)**	**0–29**	98 (12.2)	49 (10.7)	12 (9.1)	0.2
	**30–39**	185 (23.1)	107 (23.4)	18 (13.6)	
	**40–44**	134 (16.7)	79 (17.3)	23 (17.4)	
	**45–49**	143 (17.9)	84 (18.4)	31 (23.5)	
	**50–59**	188 (23.5)	112 (24.5)	37 (28.0)	
	**60+**	53 (6.6)	26 (5.7)	11 (8.4)	
**Gender**	**Male**	662 (82.6)	369 (80.7)	110 (83.3)	0.5
	**Female**	139 (17.4)	88 (19.3)	22 (16.7)	
**Nationality**	**Local**	691 (86.3)	418 (91.5)	122 (92.4)	0.7
	**Foreign**	110 (13.7)	39 (8.5)	10 (7.6)	
**Year of diagnosis**	**2013–2014**	243 (30.3)	143 (31.3)	47 (35.6)	<0.001
	**2015–2016**	177 (22.2)	86 (18.8)	36 (34.9)	
	**2017–2019**	211 (26.3)	128 (28.0)	32 (24.2)	
	**2020–2022**	170 (21.2)	100 (21.9)	7 (5.3)	

HCV, hepatitis C virus.

a Overall includes all study population, including those who were not tested for HCV RNA.

### Treatment outcome measures

Among patients with positive HCV RNA (n = 457), treatment was initiated in 239 (52.3%) cases (Supplementary Table S2). Cases of age-groups 30-39 years (adjusted odds ratio [aOR] = 2.41 95% confidence interval [CI] 1.17, 5.07]), 40-44 years (aOR = 2.55, 95% CI 1.19, 5.58), and 50-54 years (aOR = 2.76, 95% CI 1.25, 6.24)) had higher odds of starting HCV treatment when compared with those <30 years old (Table 2). Locals had 2.42 times higher odds of starting treatment for HCV compared with foreign residents (aOR = 2.42, 95% CI 1.16, 5.36). HCV cases diagnosed in 2020-2022 had lower odds of starting treatment (aOR = 0.29, 95% CI 0.16, 0.51]) compared with cases diagnosed in 2013-2014.

**Table 2:** Factors associated with HCV treatment initiation among HCV cases with detectable HCV RNA.

		Treatment initiated (n = 239) n (%)	Treatment not initiated (n = 218) n (%)	Crude OR (95% CI)	aAdjusted OR (95% CI)
**Age group (years)**	**0–29**	18 (7.5)	31 (14.2)	1	1
	**30–39**	60 (25.1)	47 (21.6)	**2.20 (1.11, 4.47)**	**2.41 (1.17, 5.07)**
	**40–44**	48 (20.1)	31 (14.2)	**2.67 (1.29, 5.65)**	**2.55 (1.19, 5.58)**
	**45–49**	49 (20.5)	35 (16.1)	**2.41 (1.18, 5.05)**	2.11 (1.00, 4.53)
	**50–54**	38 (15.9)	28 (12.8)	**2.34 (1.10, 5.06)**	**2.76 (1.25, 6.24)**
	**≥55**	26 (10.9)	46 (21.1)	0.97 (0.46, 2.08)	1.10 (0.50, 2.45)
**Gender**	**Male**	201 (84.1)	168 (77.1)	1.57 (0.99, 2.53)	1.46 (0.88, 2.45)
	**Female**	38 (15.9)	50 (22.9)	1	1
**Nationality**	**Local**	228 (95.4)	190 (87.2)	**3.05 (1.52, 6.56)**	**2.42 (1.16, 5.36)**
	**Foreign**	11 (4.6)	28 (12.8)	1	1
**Year of diagnosis**	**2013–2014**	88 (36.8)	55 (25.2)	1	1
	**2015–2016**	55 (23.0)	31 (14.2)	1.11 (0.64, 1.94)	1.13 (0.63, 2.03)
	**2017–2019**	65 (27.2)	63 (28.9)	0.64 (0.40, 1.04)	0.65 (0.39, 1.08)
	**2020–2022**	31 (13.0)	69 (31.7)	0.28 (0.16, 0.48)	**0.29 (0.16, 0.51)**

CI, confidence interval; HCV, hepatitis C virus; OR, odds ratio.

a OR adjusted with all factors presented in the table.

Among cases who initiated treatment (n = 239), 166 (69.5%) have completed it (Supplementary Table S2). HCV cases diagnosed in 2020-2022 were found to have lower odds of completing treatment (aOR = 0.16, 95% CI 0.05, 0.56) compared with cases diagnosed in 2013-2014 (Supplementary Table S3).

Among those who completed HCV treatment (n = 166), 107 (64.5%) have achieved SVR (Supplementary Table S2). Higher odds of SVR attainment were found among HCV cases diagnosed in 2018-2022 compared with those that were diagnosed earlier in 2013-2017 (aOR = 2.60, 95% CI 1.08, 6.90, Supplementary Table S4).

In our study population (n = 801), 59 (7.4%) were observed to have HCV-related complications at baseline (Supplementary Table S2). Those aged ≥50 years had 3.32 times higher odds of having complications at baseline compared with those <50 years (aOR = 3.32, 95% CI 1.93, 5.79, Table 3).

**Table 3:** Factors associated with the presence of HCV-related complications at baseline and HCV-related deaths.

		Had HCV-related complications at baseline (n = 801)				Died due to HCV (n = 129)			
		Yes (n = 59) n (%)	No (n = 742) n (%)	Crude OR (95% CI)	Adjusted OR (95% CI)	Yes (n = 47) n (%)	No (n = 82) n (%)	Crude OR (95% CI)	Adjusted OR (95% CI)
**Age group (years)**	**<50**	25 (42.4)	535 (72.1)	1	1	17 (36.2)	31 (37.8)	1	1
	**≥50**	34 (57.6)	207 (27.9)	**3.51 (2.05, 6.09)**	**3.32 (1.93, 5.79)**	30 (63.8)	51 (62.2)	1.07 (0.51, 2.28)	0.96 (0.44, 2.12)
**Gender**	**Male**	44 (74.6)	618 (83.3)	0.59 (0.32, 1.12)	0.72 (0.39, 1.39)	30 (63.8)	59 (72.0)	0.69 (0.32, 1.49)	0.66 (0.29, 1.48)
	**Female**	15 (25.4)	124 (16.7)	1	1	17 (36.2)	23 (28.0)	1	1
**Nationality**	**Local**	55 (93.2)	636 (85.7)	2.29 (0.92, 7.68)	2.06 (0.81, 6.98)	45 (95.7)	80 (97.6)	0.56 (0.07, 4.82)	0.51 (0.06, 4.46)
	**Foreign**	4 (6.8)	106 (14.3)	1	1	2 (4.3)	2 (2.4)	1	1
**Year of diagnosis**	**2013–2017**	34 (57.6)	450 (60.6)	1	1	35 (74.5)	63 (76.8)	1	1
	**2018–2022**	25 (42.4)	292 (39.4)	1.13 (0.66, 1.93)	1.12 (0.64, 1.92)	12 (25.5)	19 (23.2)	1.14 (0.49, 2.59)	1.12 (0.47, 2.60)

CI, confidence interval; HCV, hepatitis C virus; OR, odds ratio.

Among cases who died (n = 129), 47 (36.4%) were found to be related to HCV (Supplementary Table S2). Among the HCV-related deaths, 12 (25.5%) started treatment, 5 (12.6%) completed treatment, while 2 (4.3%) were cured (Figure 1). Twenty-one (44.7%) of the HCV-related deaths had HCV-related complications at baseline (Figure 1). We did not observe any association between socio-demographic factors and HCV-related deaths (Table 3). Supplementary Table 5 shows the counts and causes of non-HCV-related deaths among HCV cases.

## Discussion

Our study findings revealed three key points highlighting the need for better HCV control in Brunei Darussalam. First, the increasing prevalence suggests that the number of cases living with chronic HCV infection in the country is increasing. Second, we identified a significant proportion of HCV cases who were not tested for HCV RNA, the step needed to initiate treatment in the first place. Third, we noted the increased mortality among those with HCV-related complications at baseline.

First, the increasing prevalence observed could be due to the decreasing trend in both HCV treatment initiation and completion during the study period. This trend could be partly explained by the administrative-related barriers for patients to receive their medications. In Brunei, pegylated interferon-alpha was the primary treatment option for HCV until 2018, when DAA was first introduced. Both therapies were listed under a named-patient basis (NPB) list, leading to delays before being able to dispense the medications to the patient. In addition, medication availability was not consistent due to internal procurement processes. The inadvertent delays and disruptions in treatment could have resulted in a negative perception of healthcare providers by patients, leading to distrust and subsequent default and loss to follow-up [18,19]. Although DAA was removed from the NPB list since 2020 (thus making it more accessible), this coincided with the COVID-19 pandemic, which affected routine health care services (including patient follow-up), and could have further reduced the HCV treatment initiation and completion rates during this time [20,21].

Previous studies reported HCV treatment initiation rates among HCV RNA-positive patients ranging from 20% to over 60%, while we reported a rate of around 50% [[22], [23], [24]]. Probable reasons identified by these studies include access to care, treatment affordability, whether treatment was received at either primary care or gastroenterology clinic setting, and changes in HCV treatment policies. Although our study did not evaluate these factors, we found that HCV patients aged <55 years generally had higher odds of starting treatment. This could be due to the younger patients having fewer underlying health conditions, making them more suitable treatment candidates with fewer concerns of adverse effects when compared with older patients [25,26]. About 30% of our study population were aged ≥50 years, and this group had higher odds of having HCV-related complications at baseline. The latter could have hindered them from initiating treatment, particularly when pegylated interferon was the only treatment option.

In addition, trend differences were observed by year of diagnosis. Specifically, we found that the 2020-2022 period is associated with a high proportion of cases with HCV RNA detected (Table 1) and not tested (Supplementary Table S1), but low aORs for treatment initiation (Table 2) and completion (Supplementary Table S3). These findings are likely a consequence of the COVID-19 pandemic during this period, where health care services for diseases unrelated to COVID-19 were disrupted. Although less testing was conducted in general, those who did eventually get tested for HCV RNA either had high clinical suspicion for HCV or were symptomatic.

Furthermore, local cases have higher odds of initiating treatment than foreigners; a finding that was not surprising due to two reasons. First is the monetary cost. Although treatment and follow-up services are available at no cost for locals, foreign residents have to bear these costs either by themselves or by their employers. Second is the immigration law that requires all foreign employees to be terminated when they test positive for an infectious agent during required medical check-ups on arrival or upon employment contract renewal [27]. Hence, they would only have the option to undergo HCV treatment in their home countries.

Second, we observed that more than a quarter of new HCV cases were not tested for HCV RNA and that more than three-quarters of them were locals. This could be due to several factors related to the case management process and/or to the patient. For the former, patients with positive anti-HCV results could have either been missed by the attending physicians or not notified to the Brunei CDC, resulting in no follow-up arrangements. While for the latter, patients may fail to attend their medical appointments even after being followed up. This finding suggests that our reported HCV prevalence could be an under-estimate. Notably, HCV treatment for locals is free of charge, hence monetary cost is not a barrier to testing. Thus, this missed opportunity to test for HCV RNA among new HCV cases is concerning and warrants actions from both public health authorities and clinicians to ensure more stringent protocols are in place for follow-up and further testing.

Third, our study did not find any significant association between HCV-related deaths and socio-demographic factors. Other studies have previously identified clinical factors such as failure to achieve SVR, presence of severe liver disease at baseline, and HCV viraemia (detectable HCV RNA) to be significantly associated with mortality due to HCV [25,26,28]. Although these were not evaluated in our analysis, we found that almost half of our HCV-related deaths had baseline HCV-related complications, suggesting a need to promote early screening and adequate follow-up for new HCV cases with HCV-related complications.

One major strength of our study is the utilization of a national electronic health records system for case identification, in addition to the offline HCV registry. This allowed for a more comprehensive capture of all anti-HCV and HCV RNA-positive patients in Brunei, thus providing more accurate estimates of the population incidence and prevalence of HCV cases. Second, this study revealed the limitations of our offline HCV registry, as evident from the observed discrepancy in the number of cases captured from EVYDENCE compared with the HCV registry, and exposed the shortfall in mandatory reporting of HCV cases in the country. However, this provides a strong rationale for migrating to an online-based registry to achieve more comprehensive HCV case detection.

This study has several limitations. First, the prevalence rate in 2013 could not be determined, while the incidence cases could have been overestimated. During the adoption of Bru-HIMS in 2013, all new and existing HCV cases with a positive anti-HCV test result that year were captured as new. Because we could not obtain surveillance data before 2013, we were unable to differentiate between the new and existing cases in that year. Second, as data extraction was based on ICD-10 codes, the study relied heavily on the clinicians’ code inputs and the correct ICD-10 codes being entered for the intended information. Incorrect or unrecorded ICD-10 codes would lead to undercounting of patients with HCV-related complications at baseline. Thus, our results on the latter should be interpreted with caution. Third, only deaths recorded on Bru-HIMS were included in this study. Deaths from the community were not captured as these were not reflected in their clinical records; therefore, the death figures obtained from this study may be underreported. Fourth, we could not obtain data on the source of HCV acquisition and incarceration history among the HCV cases due to limitations in data extraction [18,19]. While this information could be documented in the patients’ clinical notes in Bru-HIMS, we are unable to accurately extract these notes from EVYDENCE yet. Anecdotally, it is not uncommon that the patients themselves are unaware of how they got infected, particularly those from non-high-risk groups. Another study reported some sources, such as intravenous drug use (32.0%) and dialysis (7.2%), among anti-HCV positive patients; however, the source was unknown for 58.0% of their patients [29]. Lastly, the present study did not include HCV genotypes, types of treatment, and the presence of other co-morbidities or co-infection with HIV or HBV in the analyses, which may better reflect the association with the outcomes of interest.

We have also identified several avenues for future research. First, improvement in data extraction to quantify the source of HCV transmission and access to information on whether cases are currently incarcerated would allow for future analyses of at-risk population groups and assess whether tailored HCV management is needed for these groups. Identifying the source of HCV transmission and key at-risk population groups in Brunei could allow us to determine the presence of HCV reinfection among those who have achieved SVR, another important point to consider for achieving the WHO’s HCV elimination goals [30]. Second, further investigation is needed to determine why new HCV cases could be missed for HCV RNA testing. Third, a more in-depth evaluation is warranted to explore other possible factors that may have contributed to the observed downward trends in treatment completion and initiation rates. Determining the reasons behind these issues is crucial to help identify potential barriers in HCV management so that more appropriate and applicable solutions can be implemented.

In conclusion, our study highlights a rising HCV prevalence likely due to decreasing trends in treatment initiation and completion. This, in turn, underscores gaps in our local HCV management and suggests the need to review follow-up protocols, medication procurement processes, and the supply chain. More than a quarter of new cases were not tested for HCV RNA, which may indicate a higher HCV prevalence than reported, and highlights the need for actions to improve existing guidelines for HCV RNA testing among new HCV cases. We found that nearly 50% of patients who died from HCV had complications at baseline, emphasizing the need for early screening and thorough follow-up. We recommend more comprehensive data collection and analysis in future studies to better capture at-risk population groups and formulate targeted strategies and interventions. An in-depth exploration of the challenges to HCV testing and treatment is also crucial to address potential barriers and develop effective solutions.

## Funding

This research did not receive any specific grant from funding agencies in the public, commercial, or not-for-profit sectors.

## Ethical approval

This study involves human participants and was approved by the Joint Research Ethics Committee of PAPRSB Institute of Health Science (IHSREC) and Medical and Health Research Ethics (MHREC), UBD (Reference no: UBD/PAPRSBIHSREC/2023/31). Informed consent was waived as the study involves the use of retrospectively collected patient data recorded by the Ministry of Health for investigations related to HCV (in accordance with the Infectious Disease Act).

## Author contributions

KSK and JW conceived the study. KSK analyzed the data and drafted the manuscript. KSK and LC interpreted the results. All authors read, revised, and approved the manuscript.

## Availability of data and materials

All relevant data are within the manuscript and its Supporting Information files. Restrictions apply to individual-level data due to ethical reasons.

## Declaration of competing interest

The authors have no competing interests to declare.

## Appendix Supplementary materials

Image, application 1
