# Supplementary material for: Epidemiology and control of hepatitis C virus infection in Brunei Darussalam: a retrospective cohort study
Source: IJID Reg. 2025 Nov 29;18:100818. doi: 10.1016/j.ijregi.2025.100818 (PMC12769816; doi:10.1016/j.ijregi.2025.100818)
Supplement: Supplementary file 1 [file mmc1.docx]

**Supplementary information for the manuscript entitled: “Epidemiology and Control of Hepatitis C Virus (HCV) infection in Brunei Darussalam: A retrospective cohort study”.**

**S1 Table.** Characteristics of HCV cases by HCV RNA testing status from 2013 to 2022, Brunei

| **Patient characteristics** | | **Overall (%)** | **HCV RNA tested**  **n (%)** | **HCV RNA**  **not tested**  **n (%)** | **p-value** |
| --- | --- | --- | --- | --- | --- |
| **Total** | | 801 (100) | 589 (73.5) | 212 (26.5) |  |
| **Median age (IQR ; range)** | | 44  (35 - 51; 0 - 83) | 45.0  (38-51; 0 - 77) | 41.0  (32-50; 0 - 83) | 0.001 |
| **Age group (years)** | **0-29** | 98 (12.2) | 61 (10.4) | 37 (17.5) | 0.004 |
|  | **30-39** | 185 (23.1) | 125 (21.2) | 60 (28.3) |  |
|  | **40-44** | 134 (16.7) | 102 (17.3) | 32 (15.1) |  |
|  | **45-49** | 143 (17.9) | 115 (19.5) | 28 (13.2) |  |
|  | **50-59** | 188 (23.5) | 149 (25.3) | 39 (18.4) |  |
|  | **60+** | 53 (6.6) | 37 (6.3) | 16 (7.5) |  |
| **Gender** | **Male** | 662 (82.6) | 479 (81.3) | 183 (86.3) | 0.1 |
|  | **Female** | 139 (17.4) | 110 (19.7) | 29 (13.7) |  |
| **Nationality** | **Local** | 691 (86.3) | 540 (91.7) | 151 (71.2) | <0.001 |
|  | **Foreign** | 110 (13.7) | 49 (8.3) | 61 (28.8) |  |
| **Year of diagnosis** | **2013 - 2014** | 243 (30.3) | 190 (32.2) | 53 (25.0) | 0.004 |
|  | **2015 - 2016** | 177 (22.2) | 132 (22.4) | 45 (21.2) |  |
|  | **2017 - 2019** | 211 (26.3) | 160 (27.2) | 51 (24.1) |  |
|  | **2020 - 2022** | 170 (21.2) | 107 (18.2) | 63 (29.7) |  |

IQR, Interquartile range; HCV, hepatitis C virus; RNA, ribonucleic acid.

**S2 Table. Characteristics of HCV cases from 2013 to 2022 by five measures of treatment outcomes: Treatment initiation, treatment completion, SVR achieved, presence of HCV-related complications at baseline, and HCV-related deaths.**

|  | | **HCV treatment initiated (n=457)** | | **HCV treatment completed (n=239)** | | **SVR achieved (n=166)** | | **Had HCV-related complications at baseline (n=801)** | | **Death due to HCV (n=129)** | |
| --- | --- | --- | --- | --- | --- | --- | --- | --- | --- | --- | --- |
| **Patient characteristics** | | **Yes (%)** | **No (%)** | **Yes (%)** | **No (%)** | **Yes (%)** | **No (%)** | **Yes (%)** | **No (%)** | **Yes (%)** | **No (%)** |
| Total | | 239 (52.3) | 218 (47.7) | 166 (69.5) | 73 (30.5) | 107 (64.5) | 59 (35.5) | 59 (7.4) | 742 (92.6) | 47 (36.4) | 82 (63.6) |
| Mean age (SD) | | 43.3 (9.48) | 44.3 (12.54) | 43.0 (8.70) | 44.1 (11.09) | 42.7 (7.97) | 43.7 (9.93) | 51.1 (9.46) | 42.7 (12.16) | 52.1 (9.13) | 53.6 (11.46) |
| Median age (IQR) | | 44.0 (37-50) | 44.5 (35-53) | 44.0 (37-49) | 44.0 (37-51) | 44.0 (38-47) | 45.0 (38-52) | 51.0 (44.5-57) | 43.0 (35-50) | 53.0 (45.5-56.5) | 52.5 (46-58.8) |
| Age range (years) | | 17.0 to 75.0 | 6.0 to 77.0 | 20.0 to 62.0 | 17.0 to 75.0 | 21.0 to 62.0 | 20.0 to 62.0 | 27.0 to 78.0 | 0.0 to 83.0 | 39.0 to 78.0 | 30.0 to 83.0 |
| Age group (years) | 0-29 | 18 (7.5) | 31 (14.2) | 11 (6.6) | 7 (9/6) | 6 (5.6) | 5 (8.5) | 1 (1.7) | 97 (13.1) | 0 (0.0) | 0 (0.0) |
|  | 30-39 | 60 (25.1) | 47 (21.6) | 43 (25.9) | 17 (23.3) | 28 (26.2) | 15 (25.4) | 4 (6.8) | 181 (24.4) | 1 (2.1) | 8 (9.8) |
|  | 40-44 | 48 (20.1) | 31 (14.2) | 34 (20.5) | 14 (19.2) | 27 (25.2) | 7 (11.9) | 10 (16.9) | 124 (16.7) | 7 (14.9) | 7 (8.5) |
|  | 45-49 | 49 (20.5) | 35 (16.1) | 39 (23.5) | 10 (13.7) | 26 (24.3) | 13 (22.0) | 10 (16.9) | 133 (17.9) | 9 (19.2) | 16 (19.5) |
|  | 50-59 | 57 (23.9) | 55 (25.2) | 37 (22.3) | 20 (27.4) | 19 (17.8) | 18 (30.5) | 24 (40.8) | 164 (22.1) | 22 (46.8) | 32 (39.0) |
|  | 60+ | 7 (2.9) | 19 (8.7) | 2 (1.2) | 5 (6.8) | 1 (0.9) | 1 (1.7) | 10 (16.9) | 43 (5.8) | 8 (17.0) | 19 (23.2) |
| Gender | Male | 201 (84.1) | 168 (77.1) | 142 (85.5) | 59 (80.8) | 94 (87.9) | 48 (81.4) | 44 (74.6) | 618 (83.3) | 30 (63.8) | 59 (72.0) |
|  | Female | 38 (15.9) | 50 (22.9) | 24 (14.5) | 14 (19.2) | 13 (12.1) | 11 (18.6) | 15 (25.4) | 124 (16.7) | 17 (36.2) | 23 (28.0) |
| Nationality | Local | 228 (95.4) | 190 (87.2) | 161 (97.0) | 67 (91.8) | 103 (96.3) | 58 (98.3) | 55 (93.2) | 636 (85.7) | 45 (95.7) | 80 (97.6) |
|  | Foreign | 11 (4.6) | 28 (12.8) | 5 (3.0) | 6 (8.2) | 4 (3.7) | 1 (1.7) | 4 (6.8) | 106 (14.3) | 2 (4.3) | 2 (2.4) |
| Ethnicity | Malay | 99 (41.4) | 96 (44.0) | 117 (70.5) | 53 (72.6) | 75 (70.1) | 42 (71.1) | 44 (74.5) | 486 (65.5) | 34 (72.3) | 61 (74.4) |
|  | Chinese | 101 (42.3) | 97 (44.5) | 18 (10.8) | 1 (1.4) | 10 (9.3) | 8 (13.6) | 4 (6.8) | 50 (6.7) | 2 (4.3) | 7 (8.5) |
|  | Others | 2 (0.8) | 3 (1.4) | 10 (6.0) | 2 (2.7) | 9 (8.4) | 1 (1.7) | 5 (8.5) | 73 (9.8) | 2 (4.3) | 4 (4.9) |
|  | Unknown | 37 (15.5) | 22 (10.1) | 21 (12.7) | 17 (23.3) | 13 (12.2) | 8 (13.6) | 6 (10.2) | 133 (18.0) | 9 (19.1) | 10 (12.2) |
| District of residence | Brunei-Muara | 88 (36.8) | 55 (25.2) | 76 (45.8) | 23 (31.5) | 52 (48.6) | 24 (40.7) | 27 (45.8) | 332 (44.7) | 19 (40.4) | 40 (48.8) |
|  | Belait | 55 (23.0) | 31 (14.2) | 64 (38.5) | 37 (50.7) | 41 (38.3) | 23 (39.0) | 28 (47.4) | 301 (40.6) | 23 (49.0) | 33 (40.2) |
|  | Temburong | 65 (27.2) | 63 (28.9) | 1 (0.6) | 1 (1.4) | 1 (0.9) | 0 (0.0) | 0 (0.0) | 11 (1.5) | 0 (0.0) | 1 (1.2) |
|  | Tutong | 31 (13.0) | 69 (31.7) | 25 (15.1) | 12 (16.4) | 13 (12.2) | 12 (20.3) | 4 (6.8) | 98 (13.2) | 5 (10.6) | 8 (9.8) |
| Year of diagnosis | 2013-2014 | 88 (36.8) | 55 (25.2) | 72 (43.4) | 16 (21.9) | 38 (35.5) | 34 (57.6) | 12 (20.3) | 231 (31.1) | 21 (44.7) | 40 (48.7) |
|  | 2015-2016 | 55 (23.0) | 31 (14.2) | 43 (25.9) | 12 (16.4) | 31 (29.0) | 12 (20.4) | 11 (18.6) | 166 (22.4) | 11 (23.4) | 18 (22.0) |
|  | 2017-2019 | 65 (27.2) | 63 (28.9) | 45 (27.1) | 20 (27.4) | 33 (30.8) | 12 (20.4) | 26 (44.1) | 185 (24.9) | 10 (21.3) | 15 (18.3) |
|  | 2020-2022 | 31 (13.0) | 69 (31.7) | 6 (3.6) | 25 (34.3) | 5 (4.7) | 1 (1.6) | 10 (17.0) | 160 (21.6) | 5 (10.6) | 9 (11.0) |

**S3 Table. Factors associated with HCV treatment completion among cases who initiated HCV treatment.**

|  | | **Treatment completed (n=166)**  **n (%)** | **Treatment not completed (n=43) n (%)** | **Crude OR (95% CI)** | ***Adjusted OR (95% CI)** |
| --- | --- | --- | --- | --- | --- |
| **Age group (years)** | **0-29** | 11 (6.6) | 4 (9.3) | 1 | 1 |
|  | **30-39** | 43 (25.9) | 10 (23.2) | 1.56 (0.37, 5.73) | 1.79 (0.40, 7.00) |
|  | **40-44** | 34 (20.5) | 7 (16.3) | 1.77 (0.40, 7.08) | 1.88 (0.41, 7.95) |
|  | **45-49** | 39 (23.5) | 6 (14.0) | 2.36 (0.53, 9.86) | 2.27 (0.49, 9.76) |
|  | **≥50** | 39 (23.5) | 16 (37.2) | 0.89 (0.23, 3.87) | 1.06 (0.25, 3.85) |
| **Gender** | **Male** | 142 (85.5) | 36 (83.7) | 1.15 (0.22, 3.04) | 1.09 (0.39, 2.80) |
|  | **Female** | 24 (14.5) | 7 (16.3) | 1 | 1 |
| **Nationality** | **Local** | 161 (97.0) | 40 (93.0) | 2.42 (0.48,10.26) | 2.38 (0.43,11.13) |
|  | **Foreign** | 5 (3.0) | 3 (7.0) | 1 | 1 |
| **Year of diagnosis** | **2013-2014** | 72 (43.4) | 13 (30.2) | 1 | 1 |
|  | **2015-2016** | 43 (25.9) | 10 (23.3) | 0.78 (0.31, 1.96) | 0.81 (0.32, 2.11) |
|  | **2017-2019** | 45 (27.1) | 12 (27.9) | 0.68 (0.28, 1.63) | 0.72 (0.30, 1.78) |
|  | **2020-2022** | 6 (3.6) | 8 (18.6) | **0.14 (0.04, 0.45)** | **0.16 (0.05, 0.56)** |

*Odd ratios (OR) adjusted with all factors presented in the table. HCV, hepatitis C virus.

**S4 Table. Factors associated with SVR attainment among patients who completed HCV treatment.**

|  | | **SVR achieved (n=107)**  **n (%)** | **SVR not achieved (n=59)**  **n (%)** | **Crude OR (95% CI)** | ***Adjusted OR (95% CI)** |
| --- | --- | --- | --- | --- | --- |
| **Age group (years)** | **0-29** | 6 (5.6) | 5 (8.5) | 1 | 1 |
|  | **30-39** | 28 (26.2) | 15 (25.4) | 1.56 (0.39, 6.03) | 1.16 (0.28, 4.62) |
|  | **40-44** | 27 (25.2) | 7 (11.9) | 3.21 (0.74, 14.12) | 2.35 (0.52, 10.65) |
|  | **45-49** | 26 (24.3) | 13 (22.0) | 1.67 (0.41, 6.59) | 1.42 (0.35, 5.69) |
|  | **≥50** | 20 (18.7) | 19 (32.2) | 0.88 (0.22, 3.39) | 0.60 (0.14, 2.42) |
| **Gender** | **Male** | 94 (87.9) | 48 (81.4) | 1.66 (0.68, 3.98) | 1.61 (0.62, 4.14) |
|  | **Female** | 13 (12.1) | 11 (18.6) | 1 | 1 |
| **Nationality** | **Local** | 103 (96.3) | 58 (98.3) | 0.44 (0.02, 3.09) | 0.41 (0.02, 3.46) |
|  | **Foreign** | 4 (3.7) | 1 (1.7) | 1 | 1 |
| **Year of diagnosis** | **2013-2017** | 79 (73.8) | 51 (86.4) | 1 | 1 |
|  | **2018-2022** | 28 (26.2) | 8 (13.6) | 2.26 (0.99, 5.66) | **2.60 (1.08, 6.90)** |

*Odd ratios (OR) adjusted with all factors presented in the table. HCV, hepatitis C virus; SVR, sustained virologic response.

**S5 Table. Details on the causes of non-HCV-related deaths for HCV cases from 2013 to 2022, Brunei**

| **Cause of Death** | **No of cases** |
| --- | --- |
| Acute coronary syndrome | 13 |
| Cerebral event (e.g. massive intracranial haemorrhage) | 4 |
| Complications from non-liver malignancy | 9 |
| Severe sepsis with multi-organ dysfunction from causes such as chest infection, foot gangrene, central line infection | 28 |
| End-stage renal disease (ESRD)-related complications | 13 |
| Trauma | 2 |
| Unknown | 13 |
| **Total non-HCV-related deaths** | **82** |
